# A glance at the actual role of glutamine metabolism in thyroid tumorigenesis

**DOI:** 10.17179/excli2021-3826

**Published:** 2021-07-12

**Authors:** Raziyeh Abooshahab, Kourosh Hooshmand, Fatemeh Razavi, Crispin R. Dass, Mehdi Hedayati

**Affiliations:** 1 Cellular and Molecular Endocrine Research Center, Research Institute for Endocrine Sciences, Shahid Beheshti University of Medical Sciences, Tehran, Iran; 2 Curtin Medical School, Curtin University, Bentley 6102, Australia; 3 Steno Diabetes Center Copenhagen, Gentofte, Denmark; 4 Department of Biology, Science and Research Branch, Islamic Azad University, Tehran, Iran; 5 Curtin Health Innovation Research Institute, Bentley, 6102, Australia

**Keywords:** thyroid cancers, metabolomics, metabolism, glutamine, amino acids

## Abstract

Thyroid cancers (TCs) are the most prevalent malignancy of the endocrine system and the seventh most common cancer in women. According to estimates from the Global Cancer Observatory (GCO) in 2020, the incidence of thyroid cancer globally was 586,000 cases. As thyroid cancer incidences have dramatically increased, identifying the most important metabolic pathways and biochemical markers involved in thyroid tumorigenesis can be critical strategies for controlling the prevalence and ultimately treatment of this disease. Cancer cells undergo cellular metabolism and energy alteration in order to promote cell proliferation and invasion. Glutamine is one of the most abundant free amino acids in the human body that contributes to cancer metabolic remodeling as a carbon and nitrogen source to sustain cell growth and proliferation. In the present review, glutamine metabolism and its regulation in cancer cells are highlighted. Thereafter, emphasis is given to the perturbation of glutamine metabolism in thyroid cancer, focusing on metabolomics studies.

## Abbreviations

KG α-ketoglutarate

TCA tricarboxylic acid

OAA oxaloacetate

GSH Glutathione

GLUD glutamate dehydrogenase

GOT glutamate oxaloacetate transaminase

GPT glutamate pyruvate transaminase

ATC anaplastic thyroid cancer

FNAB fine-needle aspiration biopsy

CITED1 Cbp/p300-interacting transactivator1

TTF-1 thyroid transcription factor 1

CEA carcinoembryonic antigen

HBME-1 hector battifora mesothelial-1

ATP adenosine triphosphate

Gln glutamine

NEAA non-essential amino acid

ASCT2 amino acid transporter-2

GLS glutaminase

mTORC1 mammalian targets rapamycin complex 1

T_3_ Triiodothyronine

GSSG oxidized glutathione 

## Introduction

Globally, thyroid cancers (TCs) are the most common head and neck malignancy and the seventh most prevalent cancer in women (Aschebrook-Kilfoy et al., 2013[4]). Histologically, TCs are classified into four major types, including papillary thyroid cancer (PTC), follicular thyroid cancer (FTC), medullary thyroid cancer (MTC), and anaplastic thyroid cancer (ATC) (Cooper, 2009[15]). Several risk factors associated with TCs have been previously determined, including a history of goiter, benign nodules, and genetic and epigenetic changes (such as mutations or aberrant methylation in multiple proto-oncogenes and tumor suppressor genes, which lead to their gain in function and loss of function, respectively) (Lemoine et al., 1988[51]) and exposure to radiation in childhood (Sarne and Schneider, 1996[66]). Today, ultrasound-guided fine-needle aspiration biopsy (FNAB) followed by cytological evaluation is considered an effective method for diagnosing thyroid nodules (Hay et al., 1992[39]). However, the main impediment to this approach is that roughly 15-30 % of all FNABs have low diagnostic accuracy and cannot discriminate malignant from benign lesions (Hedayati et al., 2020[40]). Hence, the results affect clinical decisions and remain as “indeterminate thyroid lesions” (Yassa et al., 2007[87]). Accordingly, thyroid FNABs must be repeated to prevent false-positive or false-negative results (Bongiovanni et al., 2012[7]). In some instances, lobectomy or thyroidectomy is an unavoidable choice for diagnosis and treatment (Ho et al., 2014[41]). Nowadays, the analysis of BRAF and RAS mutations is performed on FNAB samples to refine FNAB cytology results' accuracy (Su et al., 2016[74]). Other promising markers were introduced in various studies performing immunocytochemical analysis and other genetic tests as complements to FNAB cytology analysis of indeterminate lesions (Eszlinger et al., 2014[32]; Fischer and Asa, 2008[33]; Serra and Asa, 2008[69]; Yu et al., 2012[90]). Despite noticeable advances in diagnostic tools and targeted therapeutics, there are no practical diagnosis and treatment strategies for thyroid nodules/cancer. Hence, research is directed towards discovering unique diagnostic and prognostic biomarkers and ultimately find potential therapeutic targets for TCs. 

The complexity of cancer in terms of diverse processes that drive the formation, growth, development, and progression of tumor cells continues to present a great challenge to the medical field. Over the past decade, many theories of the molecular mechanisms involved in cancer with a focus on individual factors and processes have emerged. Presently, several defined hallmarks for the cancer process are co-opted together to provide a conceptual framework for the complexity inherent in cancer (Fouad and Aanei, 2017[35]). Metabolic reprogramming is one of the critical hallmarks of the cancer process, made prominent by the Warburg effect (Liberti and Locasale, 2016[52]). This phenomenon is associated with cellular metabolic changes in the aerobic glycolysis pathway, favoring a rapid supply of adenosine triphosphate (ATP) to support tumor cells' growth and proliferation (Fouad and Aanei, 2017[35]; Warburg, 1956[81]; Warburg and Minami, 1923[82]).

Glutaminolysis is another considerable metabolic reprogramming of cancer cells that mediates anaplerotic reactions to supply tricarboxylic acid (TCA) cycle intermediates (DeBerardinis and Cheng, 2010[25]). Glutamine (Gln) is a non-essential amino acid (NEAA) abundant in the human body that can be transported into cells via the amino acid transporter-2 (ASCT2), allowing Gln to enter the glutaminolysis pathway. In this process, Gln is first converted to glutamate by the action of glutaminase 1 (GLS1) (Curthoys and Watford, 1995[21]). Glutamate dehydrogenase (GDH) then converts glutamate to α-ketoglutarate (aKG), shuttled to the Krebs cycle (Curi et al., 2007[20]; Curthoys and Watford, 1995[21]). In addition, glutamate can be converted to other amino acids, including alanine and aspartate, by the action of glutamate oxaloacetate transaminase (GOT) and glutamate pyruvate transaminase (GPT), respectively (Sookoian and Pirola, 2012[73]). Gln plays a vital role in metabolism and contributes to the biosynthesis of proteins, lipids, glutathione (GSH) (to maintain redox balance), nucleotides, and NEAAs (Cluntun et al., 2017[14]; Yoo et al., 2020[88]). In thyroid cancer, the alteration of Gln levels and changes in Gln metabolism-related protein expression were reported to be directly and indirectly associated with tumorigenesis (Kim et al., 2016[47]; Yu et al., 2018[91]). 

This paper will review the experiments conducted on the role of Gln metabolism in TCs with additional consideration on possible mechanisms linking perturbation of Gln metabolism to pathological conditions.

## Glutamine Metabolism; from Normal to Cancer Cells

Gln, as an NEAA, is a neutral amino acid with an amine functional group and a 146.14 g/mol molecular weight, designated as a conditionally essential amino acid (Lacey and Wilmore, 1990[50]; Smith, 1990[72]). Gln among the twenty different amino acids has long been documented to play critical roles in cell proliferating and survival, that first was postulated by Ehrensvärd et al. (Ehrensvärd and Fischer, 1949[30]), after which it was highly detailed by Eagle et al. in 1956 (Eagle et al., 1956[29]). Gln acts as a ready carbon source in the body for producing energy aligned with glucose and fatty acids. Moreover, it can be converted to other NEAAs and glucose while facilitating the exchange of nitrogen through the transportation of ammonia from tissues in biosynthetic reactions and contributing to redox homeostasis (Labow and Souba, 2000[49]; Smith, 1990[72]). Gln and Gln-derived metabolites can be consumed by cells as a substrate for peptide and protein synthesis, purines and pyrimidine generation to nucleotide synthesis, maintain optimal antioxidant states, and regulate additional signaling pathways (Smith, 1990[72]). It is well estimated that Gln synthesis in a normal person is about 40-80 g per day (Curthoys and Watford, 1995[21]). Its production is due to the combination of glutamate and NH_4_^+^ and can occur in several tissues such as the brain, liver, lungs, adipose and skeletal muscles (Berg et al., 2007[5]). Furthermore, in human blood plasma samples derived at the fasting state, Gln concentration ranges from 500 µM/L to 800 µM/L (Psychogios et al., 2011[63]; Roth, 2008[64]). This ratio depends on certain enzymes' functions and glutamine transporters, influencing the Gln metabolism during physiological and pathophysiological conditions (Cruzat et al., 2018[16]). 

Gln metabolism and catabolism occur upon the action of two main enzymes, namely Gln synthetase (GS) and glutaminase. GS in the cytosol is subject to transform glutamate plus NH_4_^+^ into Gln and ATP products (Cluntun et al., 2017[14]; Krebs, 1935[48]). In fact, Gln can be synthesized endogenously from the conversion of α-ketoglutarate (an intermediate in the TCA cycle) into glutamate utilizing NADPH by glutamate dehydrogenase or formed by leucine catabolism which provides nitrogen atoms for the reaction (Curi et al., 2016[19]; Neu et al., 1996[57]). It is then taken up by cells and consumed according to cellular needs. Glutaminase is another main enzyme on the path of Gln metabolism, encoded by two paralogous genes in mammals, kidney-type glutaminase (GLS) and liver-type glutaminase (GLS2) (Altman et al., 2016[3]). GLS in the mitochondria is responsible for the hydrolysis of Gln to glutamate and NH_4_^+^. Moreover, by Gln breaking down, NADPH is produced, responsible for maintaining the redox state of the cells and protecting them against oxidative stress (Altman et al., 2016[3]; Curthoys and Watford, 1995[21]).

**Figure 1:**
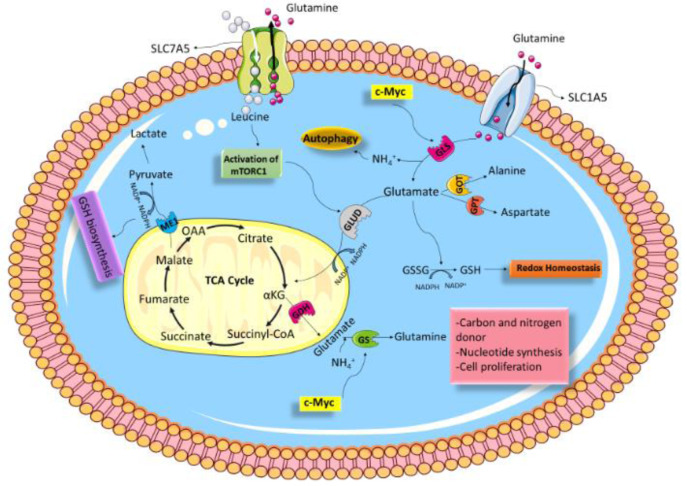
Schematic of glutamine metabolism. Various aspects of Gln metabolism are shown. Gln is taken up via SLC1A5 and enters into the glutaminolysis pathway, then is converted to glutamate via GLS. GLUD then converts glutamate to αKG, which is shuttled to the Krebs cycle. In addition, glutamate can be converted to other amino acids, including alanine and aspartate, by the action of GOT and GPT, respectively. Glutamate produced from the glutaminolysis pathway via enzymatic reaction can be used as a source for GSH synthesis. Gln is transported out of cells *via* SLC7A5, and leucine enters the cell; by doing so, mTORC1 is activated. mTORC1 can stimulate Gln metabolism, therefore promotes cancer proliferation and survival. c-Myc elevates expression of GlS and GS, then Gln metabolism in cancerous cells is increased. *Abbreviations:* *α-KG*, α-ketoglutarate; TCA, tricarboxylic acid; *OAA*, oxaloacetate; *GSH*, Glutathione; *GLUD*, glutamate dehydrogenase; *GOT*, glutamate oxaloacetate transaminase; *GPT*, glutamate pyruvate transaminase.

Fourteen Gln transporters have been recognized in mammalian cells, belonging to four distinct gene families: SLC1, SLC6, SLC7, and SLC38 (Bhutia and Ganapathy, 2016[6]; Pochini et al., 2014[62]). Among them, SLC1A5 (ASCT2) and SLC7A5 (LAT1) are considered the main cellular Gln transporters (Kandasamy et al., 2018[46]). Upon entering the cell, Gln can regulate mammalian target of rapamycin complex 1 (mTORC1) activity directly or indirectly in the absence of Rag GTPases via the glutaminolysis pathway (Durán et al., 2012[28]) or acting as the critical amino acid for leucine consumption (Jewell et al., 2015[45]). Basically, mTORC1 is known to be a cell growth and metabolism modulator. Gln is transported out of cells *via* SLC7A5/ SLC3A2, and leucine enters the cell; thereby, adapting a rate-limiting step for mTORC1 ac-tivation (Figure 1(Fig. 1)) (Csibi et al., 2014[18]; Nicklin et al., 2009[58]). From another point of view, mTORC1 has a critical role in GLS regulation (Csibi et al., 2013[17]). Indeed, mTORC1 positively regulates glutamine's anaplerotic entry to the TCA cycle via GDH (Csibi et al., 2014[18]; Takahara et al., 2020[76]). Moreover, mTORC1 activation stimulates Gln uptake; however, the underlying mechanism is still poorly understood (Nicklin et al., 2009[58]). Arguably, Gln is the precursor of GSH synthesis (Bhutia and Ganapathy, 2016[6]). GSH is a low molecular weight antioxidant synthesized in eukaryotic cytosol and exists in two types; thiol-reduced (GSH) and disulfide-oxidized (GSSG) (Forman et al., 2009[34]). GSH contributes as a fundamental factor in many cellular processes, such as DNA synthesis, signal transduction, redox regulation by enhancing ROS-detoxifying enzymes to protect cells from the ROS-induced cell damages (Diaz Vivancos et al., 2010[27]; Halliwell and Gutteridge, 1989[38]; Sies, 1999[70]). Glutamate is produced from the glutaminolysis pathway via enzymatic activities of GLS1 and GLS2 and can be used as a precursor for GSH synthesis, along with cysteine, cystine, and glycine (Yoo et al., 2020[88]). Moreover, Gln supports the malic enzyme (ME) activity that catalyzes the conversion of malate to pyruvate, which concurrently generates NADPH (Michalak et al., 2015[56]). GSH can then be generated from GSSG by glutathione reductase (GR) using NADPH as the substrate (Figure 1(Fig. 1)) (Forman et al., 2009[34]; Zhang et al., 2017[93]).

Unbridled proliferation, migratory, and invasive cancerous cells' capabilities lead to increased demand for energy. In this regard, Wise et al. described that cancer cells not only show higher demand for glucose but also have a metabolic reliance on Gln for protein translation and nucleotide synthesis (Wise and Thompson, 2010[83]). Moreover, emerging evidence has suggested that the rate of Gln transportation in malignant cells is faster than in non-malignant ones (Espat et al., 1995[31]). ASCT2/SLC1A5 and ATB0+ are among the Gln-specific transporters identified to be overexpressed in numerous cancer cells (Bröer and Bröer, 2017[9]). Acting as a Na^+^-dependent symporter of Gln, SLC1A5 variant and ATB0^+ ^can sustain the Gln demand of cancer cells (Scalise et al., 2017[67]). Furthermore, ROS production in cancer cells is under rigorous control through mechanisms involving amplified GSH synthesis. It is confirmed that Gln is taken up by SLC1A5/ASCT2 in cancer cells and enters into the glutaminolysis pathway; thereby, glutamate is formed and can be used in GSH synthesis (Bhutia and Ganapathy, 2016[6]; Yang et al., 2017[86]). It should also be noted that some oncogenes regulate glutamine metabolism. Several studies thus far have linked c-Myc with Gln metabolism. Indeed, c-Myc is recognized as the main regulator of cell proliferation, promoting the expression of several metabolic genes (Dang, 1999[22]). In this regard, data from one experiment has identified that c-Myc acts as a regulator for the *GLS* gene by suppressing miR-23a and miR-23b in human P-493 B lymphoma cells and PC3 prostate cancer cells; thereby, mitochondrial glutaminase is overexpressed (Gao et al., 2009[36]). Besides, c-Myc can stimulate active demethylation of the GS promoter; consequently, its expression is increased (Bott et al., 2015[8]). Additionally, other factors like MET (HGFR) (Yuneva et al., 2012[92]), c-Jun (Lukey et al., 2016[54]), p53 (Hu et al., 2010[42]; Tajan et al., 2018[75]), K-Ras (Daye and Wellen, 2012[24]), Rb (Nicolay et al., 2013[59]), and Rho GTPases (Wang et al., 2010[79]) have been described to regulate Gln metabolism. 

It is apparent that Gln metabolism plays a vital role in tumor progression in several ways, supporting mitochondrial oxidative metabolism, providing TCA cycle and GSH intermediates and simultaneously producing NADPH (Scalise et al., 2017[67]). Although there is no appropriate discussion focusing on Gln metabolism and its perturbation in thyroid cancer, it is worth noting that thyroid hormones influence Gln release and have regulatory effects on the activity of enzymes responsible along the Gln metabolic pathway (Parry-Billings et al., 1990[61]). Triiodothyronine (T_3_) hormone produced by the thyroid gland is essential for numerous functions in the body. T3 can modulate Gln release rate from several tissues, including the liver, kidney, intestine, and immune system cells (Parry-Billings et al., 1990[61]). Nevertheless, thyroid status in Gln metabolism is debatable.

## Perturbation of Glutamine Metabolism and Risk of Thyroid Cancer

As above mentioned, due to the unrestrained proliferation of cancer cells, ATP and high energy demand increase; thereby, tumor cells show an addiction to glucose and Gln. TCs have become top of the endocrine malignancies under unpredictable fast-growing incidence tumor category in the past 30 years, especially in females (Aschebrook-Kilfoy et al., 2013[4]). Over time, extensive literature and developments in metabolomics studies have shed light on metabolic perturbation in thyroid cancer. Metabolomic approaches can recognize metabolic profile-related disease by measuring *in vivo/in vitro* metabolites (Claudino et al., 2012[13]). This approach has been used in thyroid cancer studies to find disease-specific metabolite patterns. Several untargeted metabolomics publications have appeared in recent years, documenting such metabolic changes in thyroid cancer. From them, the Warburg effect and glutaminolysis showed the main metabolic reprograming in thyroid cancer cells (Abooshahab et al., 2019[1]). However, to the best of our knowledge, very few publications address the issue of Gln metabolism. Indeed, how Gln participates in thyroid tumorigenesis and progression is still not well understood.

### Metabolomics studies

**Figure 2:**
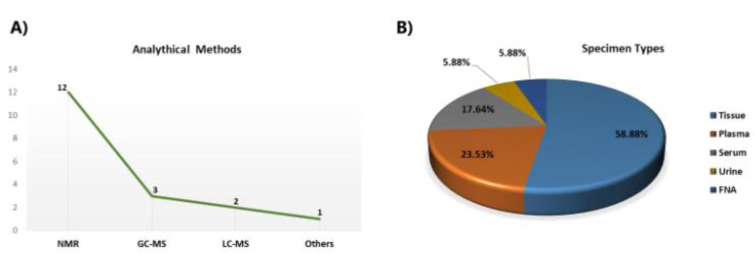
A simple descriptive summary of metabolomics studies in thyroid cancers. (A) The line graph represents the number of analytical methods, and (B) the pie chart represents the percentage of specimen types. *Abbreviations:* *FNA*, fine needle aspiration; *NMR,* nuclear magnetic resonance; *GC-MS,* gas chromatography-mass spectrometry; *LC-MS,* liquid chromatography-mass spectrometry

Most of the metabolomics studies in thyroid cancers pointed to Gln metabolism, performed by NMR spectroscopy in thyroid tissue samples (Figure 2A, B(Fig. 2)). Metabolic analysis of TCs was carried out by Torregrossa et al. using ^1^H HRMAS NMR spectroscopy to find metabolic changes in benign (nodular goiter (NG)), malignant (PTC, FTC, and ATC), and normal thyroid tissue lesions (Torregrossa et al., 2012[78]). The results showed Gln and glutamate levels were increased in tumour samples. In an article published in 2013, ^1^H-NMR-based metabolomics was used to evaluate metabolic changes in aqueous tissue extracts of healthy thyroid tissue (H), no neoplastic nodules (NN), follicular adenomas (FA), and malignant thyroid cancer [65]. In that study, glutamate was one of the remarkable elevated metabolites in all samples (Deja et al., 2013[26]). Metabolite comparison of a cohort study consisting of 1,540 serum‐plasma matched samples and 114 from healthy volunteers, benign thyroid nodule (BTN) and PTC patients, were performed by Huang et al. applying liquid chromatography‐quadrupole time‐of‐flight mass spectrometry (LC‐Q/TOF‐MS) (Huang et al., 2019[43]). D‐glutamine and D‐glutamate metabolism were observed from both serum and plasma samples as perturbed metabolic pathways in thyroid nodules. In some untargeted metabolomics studies related to thyroid cancer, researchers identified Gln/or glutamate in their study without any significant changes in their levels (Lu et al., 2016[53], Seo et al., 2018[68], Tian et al., 2015[77]). Table 1(Tab. 1) (References in Table 1: Deja et al., 2013[26]; Gu et al., 2015[37]; Lu et al., 2016[53]; Ryoo et al., 2016[65]; Seo et al., 2018[68]; Skorupa et al., 2021[71]; Tian et al., 2015[77]; Wang et al., 2020[80]; Wojtowicz et al., 2017[84]; Xu et al., 2015[85]; Zhou et al., 2020[94]) shows a summary of metabolomics studies focusing on glutamine. 

Tian et al. conducted a study to assess metabolites from thyroid lesion patients and their adjacent healthy thyroid tissues using ^1^H-NMR spectroscopy combined with GC/flame ionization detector (FID)/MS techniques (Tian et al., 2015[77]). While, Gln and glutamate were identified, no significant trends were noted among groups. Moreover, the perturbation in GSH metabolism in patients with thyroid nodules was found. In line with that, Metere et al. utilized the ^1^H-NMR spectroscopy method to identify the metabolic profile of thyroid cancer tissues (*n* = 11) in comparison with healthy thyroid tissues (*n* = 10). Their work led to the observation that the total GSH was increased in cancer lesions compared to the healthy control group (Metere et al., 2020[55]). Our team tested 55 plasma samples, including PTC (*n* = 19), MNG (*n* = 16), and healthy volunteers (*n* = 20), in a GC-MS-based metabolomics approach. It was observed that glutamic acid level was substantially increased in the PTC group compared to healthy subjects. Moreover, pathway analysis showed perturbations in D-Gln and D-glutamate and GSH metabolism with common impacts in both PTC and MNG tumorigenesis (Abooshahab et al., 2020[2]).

### Glutamine metabolism-related protein expression in thyroid cancers

In a study by Kim et al. using tissue microarray that was conducted on 557 thyroid cancer cases, including PTC: (344), FTC: (112), MTC: (70), poorly differentiated carcinoma PDC: (23), and ATC: (8), and follicular adenomas, FA (152), a distinct expression pattern in Gln metabolism-related protein among the histopathological subtypes of thyroid cancer was identified (Kim et al., 2016[47]). In that work, it was observed that ATC had a high GLS1 and GDH expression rate compared to the other subtypes. These differences can be explained in part by the characteristics of ATC. As ATC is more aggressive than the other TCs, it has a faster proliferation rate, thus subsequently shows a higher energy demand. Moreover, in contrast to other types of TCs, ATC had a link with the activation of the Wnt β-catenin signaling pathway, which regulates Gln metabolism (Oyen et al., 2007[60]; Youngblood et al., 2016[89]). On the other hand, a higher expression of human epidermal growth factor receptor 2 (HER-2) was involved in the proliferation and development of ATC (Youngblood et al., 2016[89]), which is associated with increased glutaminolysis (Cadoret et al., 2002[10]). Furthermore, GLS1 and GDH expression was elevated in PTC with the *BRAF* V600E mutation, and the expression of ASCT2 expression was raised in MTC (Kim et al., 2016[47]). Microarrays performed in 2018 by Cha et al. to evaluate the expression of glutaminolysis-related proteins in tissue samples of patients with Hürthle cell neoplasms (HCN) and follicular neoplasms (FN) (Cha et al., 2018[11]) introduced a different expression pattern for glutaminolysis-related proteins between FN and HCN, since GLS1 and GDH showed higher expression in HCN compared to FN. 

Yu et al. studied four PTC cell lines (K1, IHH4, BCPAP, and TPC-1) and tissue samples using several molecular and biochemical approaches revealing that the GLS, a key enzyme in glutaminolysis, was overexpressed in cancer specimens and played a prominent role in the development and progression of PTC (Yu et al., 2018[91]). In 2019, Chen et al. showed that overexpression of SIRT4 could inhibit Gln metabolism (Chen et al., 2019[12]). SIRT4 belongs to a family of nicotine adenine dinucleotide (NAD+)-dependent histone deacetylases called sirtuins, which have a vital contribution to energy metabolism. c-Myc oncogene is one of the principal metabolic regulators involved in glucose and Gln metabolism (Dang, 2013[23]). SIRT4 could suppress c-Myc expression (Jeong et al., 2014[44]), and in doing so, could potentially reduce Gln metabolism.

## Conclusion

At a glance, increasing evidence suggests that Gln, as a NEAA, plays an important role directly or indirectly in tumorigenesis and cancer cell survival. However, despite the emerging understanding of the biological relevance of Gln in cancer, the research underlying perturbation of Gln metabolism on TCs is restricted. From the studies mentioned above, it is evident that most of the metabolomics studies that reported changes of Gln/glutamate and related metabolic pathways were those that performed NMR approaches with untargeted strategies. Hence, it will be essential to perform quantitative and targeted assays in cohort projects to unravel the biological mechanism underlying Gln metabolism and then find the effect of other factors on it in TCs. Thyroid gland plays pivotal roles in the growth and development of the human body and keeps metabolism under control, and by releasing certain hormones, regulates Gln metabolism. Therefore, significant efforts must be made to untangle the role of Gln metabolism in thyroid tumorigenesis, which can result in a comprehensive understanding of disease progression and ultimately lead to the discovery of novel therapeutic strategies. 

## Notes

Crispin R. Dass and Mehdi Hedayati (Cellular and Molecular Endocrine Research Centre, Research Institute for Endocrine Sciences, Shahid Beheshti University of Medical Sciences, Tehran, Iran, PO Box: 19395-4763; Tel: +98(21) 22432500, Fax: +98(21) 22416264, E-mail: hedayati47@gmail.com, hedayati@endocrine.ac.ir) contributed equally as corresponding author.

## Conflict of interest

The authors declare that they have no conflict of interest.

## Authors' contributions

All authors contributed to the study conception and design. The first draft of the manuscript was written by Raziyeh Abooshahab. Kourosh Hooshmand and Fatemeh Razavi edited the manuscript. Mehdi Hedayati and Crispin R. Dass approved the final version. All authors participated in the critical revision of the manuscript for important intellectual content.

## Figures and Tables

**Table 1:**
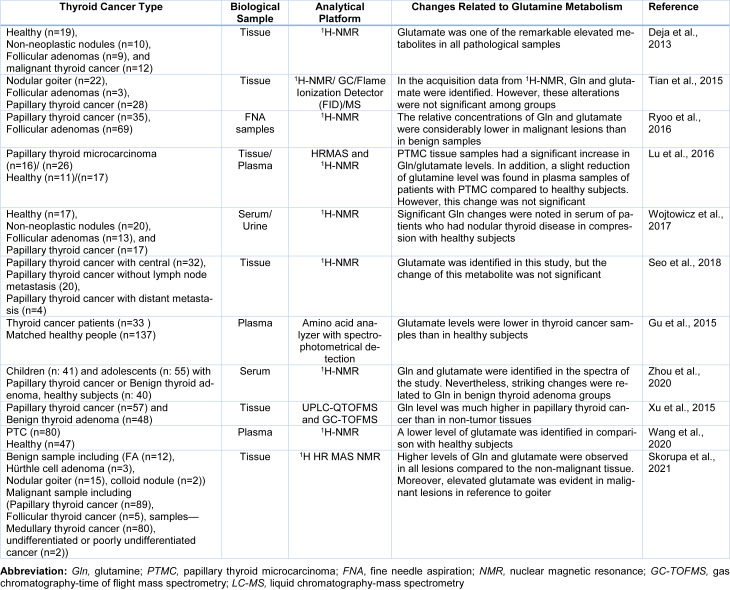
Summary of some metabolomics studies that identified glutamine/glutamate in thyroid cancer
